# Cytogenetic profile of adult AML patients in Turkey: a single center study with comprehensive comparison with literature

**DOI:** 10.4314/ahs.v22i3.21

**Published:** 2022-09

**Authors:** Ayse Cirakoglu, Rahiye Dilhan Kuru, Sukriye Yilmaz, Ayhan Deviren, Seniz Ongoren, Fevzi Firat Yalniz, Dilek Keskin, Ahmet Emre Eskazan, Ayse Salihoglu, Muhlis Cem Ar, Serdar Sahin, Yildiz Aydin, Seniha Hacihanefioglu, Zafer Baslar, Teoman Soysal, Yelda Tarkan Arguden

**Affiliations:** 1 Istanbul University-Cerrahpasa, Cerrahpasa Faculty of Medicine, Department of Medical Biology; 2 Istanbul University-Cerrahpasa, Cerrahpasa Faculty of Medicine, Internal Medicine, Division of Hematology

**Keywords:** Acute myeloid leukemia, cytogenetics, chromosomal abnormalities, adult

## Abstract

**Background:**

Cytogenetic findings are important prognostic factors in acute myeloid leukemia. Large systematic data about chromosomal characteristics of Turkish AML patients have not been reported to date.

**Objectives:**

The karyotypic profiles of 157 adult AML patients were evaluated retrospectively and compared with other reports from different populations.

**Methods:**

Cytogenetics analyses were performed on bone marrow samples using G-banding. Patients were categorized according to their cytogenetic results into four groups with the addition of a normal karyotyped group to the favorable, intermediate and adverse groups of European Leukemia Network.

**Results:**

Cytogenetic analyses were carried out successfully in 138 patients (88%). Abnormal karyotypes were found in 79 (57.2%) patients of which 13 (9.4%) were in favorable, 37 (26.8%) in intermediate and 29 (21%) in adverse groups. t(8;21) (5%) was the most common favorable abnormality while monosomal karyotypes (15.9%) in adverse group.

**Conclusion:**

This single center study is the most comprehensive study about the cytogenetic profile of acute myeloid leukemia in Turkey with comparison of other population-based studies. While there were similarities and differences with different publications, our results did not show a marked tendency to the findings of any specific geographic region.

## Introduction

Acute myeloid leukemia (AML) is a heterogeneous group of disease considering morphology, clinic, cytogenetic and molecular features. It is a malignant disorder of hematopoietic stem cells characterized by accumulation of immature progenitor cells (blasts). Genetic abnormalities are the main factors for classification of AMLs. According to the WHO classification, more than two-thirds of AML patients can be classified based on cytogenetic abnormalities and gene mutations^1^–^3^.

Despite all the advanced technologies, conventional cytogenetic is still mandatory in the assessment of AML patients^4^,^5^. Cytogenetic findings are included in the main factors by the European Leukemia Network (ELN)^6^ for risk-stratification and to classify AML cases into three groups as favorable, intermediate and adverse. According to this stratification; t(15;17), t(8;21) and inv(16)/t(16;16) are considered as favorable, whereas t(6;9), inv(3)/t(3;3), -5/del(5q), -7/del(7q), t(9;22), abn (17p), rearrangements of 11q23, complex (CK) and monosomal karyotypes (MK) are interpreted as adverse risk groups. The existence of three or more chromosomal aberrations are defined as CK^7^, and 2 or more autosomal monosomies or one single autosomal monosomy with structural abnormalities except the favorable ones are described as MK^8^. The intermediate risk group include t(3;5), t(9;11) and the other cytogenetic abnormalities which are not classified as favorable or adverse^4^,^6^,^9^.

AML can occur in all age groups, but the frequency is increased in elder adults with a median age at diagnosis of 68 years. Clonal chromosomal abnormalities are seen in more than 50% of adult AML patients^10^. Beside the rearrangements that were included in risk stratification, many other translocations and aberrations are observed^11^,^12^. Cytogenetic profile of AML patients had shown geographic heterogeneity in previous studies^13^. Incidence of chromosomal aberrations shows variation between different populations^14^–^23^.

To date, except for a few studies with limited number of cases^24^,^25^, no systematic data has been reported for AML patients in the Turkish population. In this study we aimed to present the cytogenetic characteristics and clinical features of Turkish adult patients from a single center and the comparison of our results with other population studies.

## Materials and Methods

This study includes 157 patients with de novo AML who were referred from Istanbul University-Cerrahpaşa, Cerrahpaşa Medical Faculty, Department of Internal Medicine, Division of Hematology to the Cytogenetics Laboratory of the Medical Biology Department of the same institution. Ethical approval was obtained from Ethics Committee of Istanbul University Cerrahpasa Medical Faculty.

Cytogenetic analyses were performed on bone marrow samples using standard techniques including G-banding following overnight, 24 and 48 h cultures at the time of diagnosis. Clonal chromosomal aberrations were defined according to the International System for Human Cytogenetic Nomenclature (ISCN 2016)^26^.

Presence of the same structural aberrations or gain of whole chromosomes in two or more cells and loss of chromosomes in three and more cells were defined as a clonal chromosomal abnormality.

We categorized our cases with respect to their cytogenetic results into four groups with the addition of a normal karyotyped group to the favorable, intermediate and adverse groups of ELN.

Clinical data (white blood counts (WBC) and blast counts of bone marrow were collected from clinical files of the patients.

Statistical analyses were performed using IBM SPSS Statistics 26. The age, WBC and bone marrow blast means, and remission status after induction therapy of the risk groups were analyzed separately by the Kruskal Wallis test. Dunn's Post Hoc test was used for paired comparisons between the groups for differences in multiple comparisons. Nonparametric tests were used for the low sample size below 30.

A p-value less than 0.05 was considered statistically significant for all of the tests.

## Results

Of the 157 patients, 91 (58%) were males and 66 (42%) were females with a median age of 49 (range from 18 to 89 years old). Cytogenetic analyses were carried out successfully in 138 patients (87.9%). Normal karyotype was observed in 59 (42.8 %) patients while clonal chromosomal abnormalities were detected in 79 (57.2 %). Distribution of chromosomal abnormalities into risk groups were; favorable karyotypes in 13 (9.4%), intermediate in 37 (26.8%), and adverse in 29 (21%) patients. Table 1 lists general and clinical characteristics of the patients. One hundred nineteen of the cytogenetically studied 138 cases had induction therapy, and the induction response results are summarized in Table 2. There was no statistically significant difference between the groups (p=0.534).

**Table 1:** Clinical characteristics of the risk groups

Cytogenetics	Cases (%)	Median age (Range)	Sex		WBC count (median)	Median PB blasts % (Range)	Median BM blasts % (Range)
			F	M			
Normal karyotype	59 (42.8)	45.5 (19–86)	22	37	10000 (490–238000)	40 (0–100)	68.5 (0–100)
Favorable	13 (9.4)	35 (18–62)	4	9	5900 (4000–41800)	22 (0–88)	64.5 (11–86)
Intermediate	37 (26.8)	52.5 (21–71)	15	22	37000 (6900–166000)	50 (2–100)	58.5 (10–95)
Adverse	29 (21)	49 (21–78)	15	14	11000 (1800–206800)	57.5 (0–86)	64 (25–96)
Total	138 (100)	49 (18–86)					

WBC: White Blood Cell; PB: Peripheral Blood; BM: Bone Marrow; CR: Complete Remission

**Table 2:** Induction responses of the cytogenetic risk groups.

Cytogenetic Risk Groups	CR N (%)	Non-CR N (%)	Total N (%)
Normal karyotype	31 (62)	19 (38)	50 (100)
Favorable	8 (61.5)	5 (38.5)	13 (100)
Intermediate	23 (62)	8 (38)	31 (100)
Adverse	14 (48)	11 (52)	25 (100)
P value	>0.05		

CR: Complete remission

Favorable group included seven (5%) patients with t(8;21), five patients (3.6%) with t(15;17), and one patient (0.7%) with inv(16). All the patients with t(8;21) had additional abnormalities, like del(9q), del(6q), del(1q), del(11q) and numerical abnormalities. t(15;17) was the sole abnormality in three patients, while additional abnormalities (del(6q), del(11p), del(16p) and I (17q)) were observed in the other two patients. Inversion 16 was the sole abnormality in one patient.

Of 29 patients in adverse risk group, 22 (15.9%) had MK, 20 (3.6%) had CK, two had abn(11q23) and one had t(9;22). Seventeen of 22 MKs were also CKs, so they were included in both groups. In adverse group, -5/del5 were observed in seven patient while -7/del7q in eight, and three patients had both. All the patients who had -5/del5 and -7/del7, were involved in MK group. In intermediate group, none of the patients had t(9;11) that was reported as frequent abnormality for this group. Favorable and adverse abnormalities are shown in Table 3. The other chromosomal abnormalities that detected in our cases are presented in Supplementary Table.

**Table 3:** Chromosomal abnormalities in the risk groups

Abnormality	n (%)
Favorable	
t(15;17)	5 (3.6)
t(8;21)	7 (5)
inv(16)	1 (0.7)
Adverse	
inv(3)(p21p26)*	1 (0.7)
-5/del(5q)*	10 (7.2)
-7/del(7q)*	11 (7.9)
del(11)(q23)^†^	3 (2.2)
t(9;22)	1 (0.7)
Complex Karyotype	20 (14.5)
Monosomal Karyotype	22 (15.9)

Statistical analyses for corr elations between means of white blood cell counts (WBC) and four cytogenetic risk groups showed differences between the groups (p=0.002). The post hoc test for pair wise comparison showed that WBC mean value (52839.29) of the intermediate group were higher than mean values of the three groups; normal karyotypes (37700.49), adverse (38789.13) and favorable (13860). However, there were no significant differences between WBC mean values of normal, adverse and favorable groups.

There was no statistically significant difference between the mean age of the four patient groups (p=0.210) and no significant difference in blast counts of bone marrow (p=0.444).

## Discussion

Cytogenetic analysis is one of the major prognostic indicators for AML. Chromosomal abnormalities play an important role in classifying patients into subgroups with clinical features. Despite the development of new generation molecular technologies, cytogenetic analysis remains the gold standard for AML^4^,^5^.

To date, numerous reports of cytogenetic data of different populations observed the role of geographic and ethnic variations in cytogenetic profiles of AML patients^14^–^23^,^27^. To the best of our knowledge, no systematic data has been reported about cytogenetic profile for AML patients in the Turkish population. Although this study reports the results of a single laboratory, our institution is a tertiary medical center that provides advanced medical services to the whole country. Therefore, our results can be considered representative of Turkey. The comparative analyses of the cytogenetic characteristics in adult AML patients from different populations including this present study are displayed in Table 4.

**Table 4:** Comparison of our study with population-based reports from different regions of the world

	Preiss et al (Denmark, 2003) (28)	Bacher et al (Germany, 2005) (29)	Gmidene et al (Tunisia), 2012) (19)	Gangatharan et al (Australia, 2013) (20)	Lazarevic et al (Sweden, 2014) (21)	Cheng et al (China, 2009) (18)	Grimwade et al (UK, 2010) (42)	Sierra et al (Spain, 2006) (15)	Byun et al (Korea, 2016) (22)	Udayakumar et al (Omani, 2007) (17)	Vaskova et al (Slovak, 2015) (46)	Enjeti et al (Singapore, 2004) (14)	Shaikh et al. (Pakistan, 2018) (23)	Present study (Turkey, 2020)
Number of patients	303	2555	631	898	3251	1432	5876	1129	2806	63	90	501	321	157
Median age (range)	67 (16–93)	(21–70)	37 (8 days-95 years)	66 (16–94)		42 (4–84)	44 (16–59)	61 (1–94)	51 (14–89)	25 (4–75)	54.5 (24–80)	48 (15–100)	(.15)	49 (19–89)
Normal (%)	47.0	47.7	51	38.8	43	42	41	36.5	41.4	56		39	61.1	42.8
t(15;17) (%)	3.3	5.3	10.6	7.8	excluded	14	13	14.8	8.6	6	5.5	11	4.9	3.6
t(8;21) (%)	3.3	4.1	9.4	2.5	1.9	8	7	2.7	8.8	5	4.4	7.5	8.3	5
inv(16)/t16;16) (%)	2.0	4.0	3	3.6	2.2		5	2.7	3.6	3	-	1.1	0.7	0.7
11q23 abn (%)	2.3	7.0	3.2	0.4	1.1	1	1	3.3	2.1	2	6.6	0.9		2.2
-5 (%)	5.0						2							
del5q (%)	9.9	1.2					2							
-5/del5q (%)			1.9	8.8	13	1		9.1	4.2	6	4.4	6.6		7.2
-7 (%)	8.7						5			5			1	
del7q (%)	4.7						2							
-7/del7q (%)		1.9	2.4	12.1	13	1		8.6	5.8		5.6	7		7.9
Complex (%)		12.9	8.8	13.8	24	6	14	19.3	12.5			17	9	14.5
MK (%)				13.5	18									15.9

A number of studies observed incidences of clonal chromosomal abnormalities in adult AML patients between 44.5-63.5%^15^–^23^,^28^. In this present study, clonal chromosomal abnormalities were found in 57.2% of 138 patients that were karyotyped successfully. According to their cytogenetic findings, cases were classified into four groups; normal karyotyped, favorable, intermediate, and adverse.

### Favorable Abnormalities

Favorable abnormalities were found in 10% of the patients. The most common chromosomal abnormality in favorable group was t(8;21) (5%) in our cases, although in most of the studies t(15;17) was declared as the most common favorable abnormality. The frequency of t(8;21) was reported ranged between 1.9–9.4% in previous studies^16^–^20^,^28^–^30^,^46^ (Table 4).

The frequency of t(15;17) in our study group was 3.6%, close to the results of Preiss et al who found 3.3%^28^. Many researchers reported higher rates for this abnormality between 4.9 and 14.8 % (Table 4).

Inversion (16) was observed 0.7% of our patients, which is the same as the study of Shaikh et al [23]. This frequency was reported between 1.1 and 5% by other studies (Table 4).

### Adverse Abnormalities: MKs and CKs

Our 29 (21%) patients were in adverse risk group, in which 22 (15.9%) had MK, 20 (14.5%) had CK, 2 had abn(11q23) an one had t(9;22). MKs were the most prominent abnormality (72%) in the adverse group, which constituted of 15.9% of the whole study group, in between the range of other studies which is 6–18%^21^,^31^–^33^. The unfavorable abnormalities such as inv(3)(q21q26)/t(3;3) (q21;q26), -5/del(5q), -7/del(7q), etc. were reported as associated with MKs^34^–^37^. In our cases, all of -5/del(5q) and -7/del(7q), and inv(3)(q21q26) in one case were included in MKs. Complex karyotpes were observed in 20 (14.5%) patients of adverse group.

abn(11)(q23):

Abnormalities of 11q23 other than t(9;11) were observed in 2.2% (n=3) of our patients. Our range was close to the results of Preiss et al^28^, Byun et al^22^ and Udayakumar et al^17^ who reported between 2–2.3%. Abnormalities of 11q23 were deletions in our patients while other studies observed that it was involved in balanced translocations mostly^16^–^18^,^20^,^28^,^29^. Deletions of 11q23 are associated with poor outcome in adult AML cases in the literature^38^,^39^. In one of our patients, del(11q23) were found with t(8;21) and considered in favorable group. In two patients, del(11q23) was the sole abnormality and these patients showed poor outcome and short survival.

### Intermediate Abnormalities

Chromosomal abnormalities associated with intermediate risk group were declared as t(9;11)(p21.3;q23.3) and cytogenetic abnormalities which are not classified as favorable or adverse by ELN^6^. We did not observed t(9;11), but detected other chromosomal abnormalities in our patients (Supp Table). All detected abnormalities were reported previously in AML^40^.

### Normal Karyotypes

The frequency of normal karyotypes that was 41.5% in our study. The risk stratification of AML include mutations of the genes; NMP1, FLT3, CEPBA, RUNX1, ASLX1, TP53 associated with cytogenetic abnormalities or normal karyotypes^4^,^6^,^9^. Since our study used retrospective information over a long-time span, mutation tests for these genes were not available in most of the patients. Therefore, without any knowledge about gene mutations, this group remained unclassified into risk groups.

It is considered that cytogenetic abnormalities in AML show geographic/ethnic heterogeneity^13^,^15^,^27^,^41^. In general, our results did not show a marked resemblance to any geographic region. We observed closer abnormality frequencies in one or two parameters compared with the studies from Denmark, UK (Grimwade), Korea, Omani, Singapore, Sweden, China, Slovakia, and Pakistan. Therefore, cytogenetic profile of our population cannot be considered as close to any specific geographical region. This situation probably reflects the heterogeneity of the Turkish population due to the geographical location of the country.

The median age was 49 (mean 46) in our patients. Although in most studies, the median and mean ages were reported higher than our ranges (Table 3), Enjeti et al^14^ and Grim wade et al^42^ have median and mean ages closer to ours. It was discussed that this variety between median and mean ages could either be a genuine geographic/ethnic difference or occurred due to referral biases^14^,^17^,^29^. Different studies showed association between cytogenetic aberrations and age. They concluded that unfavorable abnormalities have been observed more often in older patients^29^,^30^,^43^,^44^. We did not find significant difference between mean ages of the risk groups but that could be due to small number of cases.

We did not find any significant differences between blast counts of bone marrow, of the risk groups. After the induction therapies, CR rates were quite similar between normal karyotype (62%), favorable (61.5%), and intermediate (62%) groups, while it was lower in the adverse group (48%). But there was no statistically significant difference. These are probably because of the smallness of our case numbers.

A high white blood cell (WBC) count at the time of diagnosis was considered as another risk factor for AML and associated with poorer outcomes within favorable and intermediate risk groups^30^,^45^. In our study, comparison of cytogenetic risk groups and mean values of WBC counts showed no statistical significance between normal karyotype, favorable, and adverse groups, whereas in intermediate group's mean of WBC were higher than the other risk groups. Since we did not find any difference in induction responses between risk groups, it is not easy to interpret if this rising of WBC in intermediate group is coincidental or not.

The lack of mutation tests of AML associated genes and the low number of cases can be considered as limitations of this study.

## Conclusion

This single center study is the most comprehensive study to date showing the chromosomal characteristics of Turkish AML patients. We compared our findings with different populations from different regions of the world. While similarities and differences with different publications, our results did not show a marked tendency to any specific geographic region. More studies with larger cohorts are needed to reveal the chromosomal characteristics of Turkish AML patients.
